# The Afferent Visual Pathway: Designing a Structural-Functional Paradigm of Multiple Sclerosis

**DOI:** 10.1155/2013/134858

**Published:** 2013-10-31

**Authors:** Fiona Costello

**Affiliations:** Departments of Clinical Neurosciences and Surgery (Ophthalmology), Hotchkiss Brain Institute, University of Calgary, Canada

## Abstract

Multiple sclerosis (MS) is a disease of the central nervous system (CNS) believed to arise from a dysfunctional immune-mediated response in a genetically susceptible host. The actual cause of MS is not known, and there is ongoing debate about whether this CNS disorder is predominantly an inflammatory versus a degenerative condition. The afferent visual pathway (AVP) is frequently involved in MS, such that one in every five individuals affected presents with acute optic neuritis (ON). As a functionally eloquent system, the AVP is amenable to interrogation with highly reliable and reproducible tests that can be used to define a structural-functional paradigm of CNS injury. The AVP has numerous unique advantages as a clinical model of MS. In this review, the parameters and merits of the AVP model are highlighted. Moreover, the roles the AVP model may play in elucidating mechanisms of brain injury and repair in MS are described.

## 1. Multiple Sclerosis: An Overview

Multiple sclerosis (MS) is an inflammatory disorder of the central nervous system (CNS) which causes progressive neurological disability over time [1]. Affecting more than two million people worldwide, MS is recognized as the leading cause of nontraumatic neurological disability in young adults [2].  For many patients, clinical manifestations involve the motor, sensory, visual, and autonomic systems, but less-localizing symptoms and signs are also common, with fatigue being foremost among them [1]. The diagnosis of relapsing remitting MS (RRMS) can often be established on clinical grounds [3] for patients who experience two or more neurological events consistent with multifocal CNS inflammation. In the case of primary progressive MS (PPMS), neurological decline progressing for over a year with supporting paraclinical evidence of CNS inflammation is considered proof of the diagnosis [4–7]. Since the publication of the original McDonald criteria and subsequent iterations [5–7], radiological endpoints have been used to confirm the diagnosis of MS, in the absence of recurrent clinical events.

The majority (85%) of MS patients initially present with episodes of neurological dysfunction in the relapsing-remitting phase (RRMS) [1, 3–9], before transitioning to a secondary progressive course (SPMS) of the disease [1, 8–10]. During this time, they accumulate neurological disability with or without relapses. Approximately 15% of patients experience a primary progressive course from onset, either without preceding relapses (PPMS) or with superimposed neurological events in what is known as progressive relapsing MS [8–10]. While the acronyms RRMS, SPMS, and PPMS are embedded in the lexicon of neurologists, these labels are merely descriptors and tell us nothing about underlying differences in pathobiology that distinguish MS phenotypes. At best, they represent our clinical perceptions of different ages and stages of the disease [10]. Natural history data has shown that the progressive phase of MS in an age-dependent process [1, 9]. In the corticospinal tract, for example, chronic axonal loss, which is believed to represent the pathological substrate for disability progression, begins early in the disease course, before the expression of clinical symptoms [1, 9]. Similarly, Confavreux and Vukusic [8] have demonstrated that the time to reach disability milestones and the ages at which these landmarks are reached follow a predefined schedule that is unaffected by relapsing remitting episodes, or indeed, by the initial disease course in MS patients [1, 8]. Hence, there is evidence to suggest that MS disease progression may be governed by factors independent of inflammatory activity in the CNS. 

Currently, the driving force behind progression and the variables that affect transition from the relapsing remitting phase to the treatment-resistant progressive course in MS remain obscure. The context of this uncertainty has important implications because approved MS treatments act predominantly by targeting inflammation within the brain and spinal cord with an implicit assumption that recurrent, chronic inflammatory disease activity exacts a toll on the structural integrity and functional eloquence of the CNS over time. The purpose of this review is to discuss current perceptions regarding the pathogenesis of MS and highlight how key hypotheses might be explored using the afferent visual pathway (AVP) as a clinical model of the disease.

## 2. The Pathogenesis of Multiple Sclerosis: A Riddle Wrapped in an Enigma?

Traditionally, the mechanistic underpinnings in MS have been viewed as a deranged immune-mediated response to an environmental exposure in a genetically susceptible host [1]. The pathological “signature” of the disease is the sclerotic plaque, which is believed to represent the cumulative effects of several processes including inflammation, demyelination, remyelination, oligodendrocyte depletion, astrocytosis, axonal damage, and neuronal loss affecting white and grey matter CNS structures [1, 11]. There is evidence to suggest that neurodegeneration within these plaques forms the basis for disabling aspects of the disease [1, 11]. Yet, effector mechanisms that influence the relapsing (presumed inflammatory) and progressive (presumed neurodegenerative) phases of MS are considered to be different [1, 11]. Not surprisingly, in a condition that has chronic and fulminant forms with a wide-ranging phenotypic expression, a myriad of pathogenic mechanisms and combinations thereof have been proposed including [1, 11–13] (1) CNS inflammation as the main pathogenic event; (2) neurodegeneration as the primary event with CNS inflammation as a secondary response; (3) coexisting CNS inflammation and neurodegeneration; and (4) CNS inflammation triggering an intrinsic neurodegenerative susceptibility in a given host [1]. Similarly, numerous factors have been linked to tissue injury in MS including T-cell infiltrates and macrophage influx; antibody and complement-mediated immune reactions against oligodendrocytes and myelin; hypoxic damage; and a genetic defect or polymorphism resulting in primary susceptibility of the oligodendrocytes to immune injury [1, 11–13]. Originally, it was proposed that individuals with MS had only one type of pathological lesion, but it is now accepted that different patterns of tissue pathology can co-exist in the same patient [1]. In fact, the interplay between these proposed mechanisms has been used to explain differences in the extent of demyelination, oligodendrocyte injury, remyelination, and axonal damage seen across the spectrum of MS. Alternatively, it has been proposed that intrinsic to the disease may be T-cell-mediated brain inflammation, the manifestations of which are variably modified by different immunological effector mechanisms, resulting in what Compston [1] has elegantly described as a “state of mechanistic complexity rather than true disease heterogeneity.” While the debate about the pathogenesis of MS continues, there is at least some consensus that inflammation, neuronal loss, and axonal damage are common pathways contributing to disability in the disease. 

### 2.1. Factors Contributing to Neurological Disability in Multiple Sclerosis

#### 2.1.1. Axonal Damage

The integrity of the oligodendrocyte-myelin-axonal unit [11] is integral to function in the CNS. Myelin increases the cross-sectional diameter of the nerve axon, which improves conduction velocity and contributes to its tropic support [11]. Cytokines, nitric oxide, proteases, superoxide, CD8+ T cells, and glutamate excitotoxicity have all been shown to contribute to axonal injury [11]. While it has been known since the Charcot era that axonal injury is a feature of the MS plaque [11], understanding regarding the role of axonal loss was obscured for a period of time when demyelination was considered the predominant mechanism of injury. Interest in the impact of early axonal damage on neurological disability resurged after Trapp and colleagues [14] reported the findings from brain tissue obtained at autopsy in MS patients. Axonal transection, characterized by the presence of terminal axonal ovoids, was a prominent feature in acute and chronic lesions; the extent of axonal injury was related to the degree of inflammation [14]. At that time, Trapp et al. highlighted the need for non-invasive techniques that could be used to monitor axonal pathological changes in MS, which may be the pathological correlate of irreversible neurological impairment in the disease. Hence, any clinical model of MS should ideally provide a means of quantifying axonal damage and correlating axonal deficits with clinically relevant manifestations of the disease.

#### 2.1.2. Neuronal Loss

At this point, the cause of neuronal injury in MS is unclear [15, 16]. Neurodegeneration within the CNS may arise from retrograde axonal degeneration [16], which is viewed as a dying back phenomenon causing pathological changes in the cell body proximal to a point of injury along an axon [17]. Alternatively, anterograde or Wallerian degeneration may precipitate a dying forward process affecting the part of the axon that is separated from the cell body, which degenerates distal to the injury [17]. Transsynaptic degeneration refers to neuronal damage caused by loss of synaptic input when fibers afferent to them are injured [18]. When transsynaptic degeneration occurs, axonal atrophy is followed by neuronal cell loss and reactive gliosis [18]. The phenomenon has been well documented in the afferent visual pathway, wherein cells of the lateral geniculate bodies are known to degenerate with lesions in the retina, optic nerve, and/or optic tract. Transsynaptic degeneration may also occur in a retrograde fashion, such that lesions of the optic radiation or calcarine cortex may cause degeneration of retinal ganglion cells [18].

Recently, Green and colleagues [15] performed a large scale pathological analysis of retinal tissues in MS patients and showed that retinal involvement was extensive in the disease, with regions of retinal nuclear loss in both the ganglion and inner nuclear cell layers in MS eyes. These findings demonstrated that the retina represents an ideal substrate to determine whether neuronal pathology is related to humoral mechanisms or alternative processes [15]. Retinal ganglion cells (RGCs) are CNS neurons located within the innermost cellular layer of the retina and represent the output neurons for the deeper retinal cells (photoreceptors, horizontal cells, bipolar cells, and amacrine cells) that perform the initial phases of visual processing. Kanamori and others [17] have shown that real-time confocal scanning laser ophthalmoscopy can be used to image RGC apoptosis, intracellular signaling, and intraretinal axonal degeneration in rodents in vivo. In a recent study they used these techniques to assess the time course of Wallerian and retrograde degeneration of unmyelinated RGC axons in living rats for a month after intraretinal axotomy. It was noteworthy that the rate and magnitude of retrograde and Wallerian degeneration in this model of transection of unmyelinated CNS axons were synchronous, suggesting a common mechanism for bidirectional axonal loss after injury within the CNS [17]. These findings may be particularly germane in the setting of MS, because understanding mechanisms of axonal degeneration, both in terms of how they align and diverge with mechanisms of neurodegeneration, may be critical for developing new therapeutic interventions in this disease. 

#### 2.1.3. Remyelination

Remeylination is a prominent early feature in MS, and has been shown to be most active during the acute inflammatory process and coincide with phagocytic removal of myelin debris [11]. Oligodendrocyte precursors in the CNS that migrate to evolving MS lesions have the potential to remyelinate naked axons [19, 20]. In 20% of MS patients, plaques are eventually remyelinated, whereas in other instances remyelination is less successful [19, 20]. Remyelination restores saltatory conduction of nerve impulses and prolongs the survival of previously demyelinated axons, thereby enhancing axonal integrity [19, 20]. At the level of the cortex, remyelination is more likely to occur when there is an abundance of oligodendrocyte progenitor cells, a relative paucity of reactive astrocytes, and minimal expression of extracellular matrix molecules known to inhibit oligodendrocyte production and differentiation [20]. In a recent report, postmortem brain tissue was examined to determine the capacity for cortical lesions to remyelinate relative to adjacent subcortical white matter [20]. In demyelinated lesions, there was greater evidence of remyelination in the cortex relative to subcortical CNS regions [20]. Cortical remyelination was evident regardless of disease duration or chronological age of the patient [20]. Because remyelination indicates capacity for CNS repair, a surrogate marker for this process would be integral to any putative model of MS, particularly in clinical trials testing novel myelin repair strategies.

### 2.2. The “Outside-In” versus “Inside-Out” Debate

Dispute regarding whether MS is a primary inflammatory process with degenerative features versus a degenerative disease with inflammation occurring as an epiphenomenon has spawned ongoing debate. The “*Outside-In*” model of MS advocates that the inciting pathogenic event in MS is inflammation, with migration of autoreactive T cells across the blood-brain barrier from the systemic circulation [21]. This inflammatory influx has been viewed as a misguided immunological reaction to an instigating event perpetrated outside the CNS. While the antigen specificity of the “Outside-In” model of MS has remained unclear, myelin proteins have been regarded as the “prime suspects” [1, 21]. In support of this theory, delivery of myelin-derived proteins in adjuvant to laboratory animals has been shown to induce experimental autoimmune encephalomyelitis (EAE) [21]. Furthermore, T cells specific for myelin antigens in MS patients have been identified as being qualitatively different from those in healthy individuals [1, 21]. According to the “Outside-In” model, once an antigenic response has been triggered, there is a consequent accumulation of T and B lymphocytes, plasma cells, and macrophages and an amplification of proinflammatory cytokines through recruitment of naive microglia to create an inflammatory cascade [1]. Ultimately, when the blood-brain barrier has been breached, contact is established between activated microglia and components of the CNS oligodendrocyte-myelin unit. The inflammatory influx is mediated by T-cell lymphocytes that secrete interleukins [1], disrupt the human blood-brain barrier, and allow efficient penetration of Th17 cells into the brain, which in turn destroy neurons [1]. Integral to the “Outside-In” model is the premise that MS patients have underlying regulatory defects in their immune system that allow circulating lymphocytes to set up an immune response within the CNS [21]. Failure of regulatory mechanisms is felt to account for the sites sclerotic plaques commonly occupy in MS, which include the lateral ventricles, corpus callosum, cortex, subcortical white matter, optic nerves, brainstem, and spinal cord [21].

Like T cells, B cells are believed to have a role in perpetuating the CNS inflammation in the Outside-In model. This is inferred from the increased oligoclonal bands and immunoglobulin synthesis within the cerebrospinal fluid (CSF) of MS patients [2] and histopathological evidence of immunoglobulin deposition and complement activation in acute demyelinating lesions [2]. Until recently, however, direct proof of clinically relevant antibodies in MS had not been established, and the molecular targets for humoral responses in the disease were unknown. In 2012, Srivastava and colleagues [22] identified IgG1 and IgG3 antibodies that bind glial cells in human brain tissue in the serum of MS patients and demonstrated that the molecular target of these antibodies was the potassium channel KIR4.1. This potassium channel is expressed on astrocytes and is important for maintaining water balance [22]. Antibodies directed against KIR4.1 were detected in the serum of 186 of 397 (47%) of MS patients, relative to 1% of patients with other neurological diseases (*n* = 329). These same antibodies were absent in healthy controls [2, 22]. Injection of anti-KIR4.1 antibodies into the cisterna magna of mice was associated with reduced KIR4.1 expression in the brain, and complement activation at sites of antibody binding [2, 22]. Thus, KIR4.1 potassium channels were interpreted to be the target of an autoantibody response in some MS patients [22].

In contradistinction, the “Inside-Out” model views MS as a primarily progressive disease, which proceeds similarly to other neurodegenerative disorders, remaining relatively unaltered by excess inflammation [21]. The “Inside-Out” model argues that “cytodegeneration” of the oligodendrocyte-myelin complex is the primary pathogenic event, with inflammation occurring as a secondary response [21]. Proponents of the Inside-Out model assert that what distinguishes MS from other primary degenerative processes such as Parkinson's disease and Alzheimer's disease is the host's tendency, depending on phenotype, to react to the highly autoantigenic components that are released as a consequence of the cytodegenerative process [21]. They view MS as a “convolution” between progressive cytodegeneration and a variably primed immune system [21]. In the context of the Inside-Out model, degeneration of white and grey matter elements may proceed independently, and the extent of the secondary inflammatory reaction may be governed by the amount of released immunogenic myelin-derived material [21]. As a cytodegenerative process, MS is felt to be most “faithfully” represented in the PPMS form of the disease [21].

## 3. The Afferent Visual Pathway Model: A Functionally Eloquent and Structurally Competent Region of the Central Nervous System

Attitudes continue to evolve from viewing MS as a demyelinating disease to a broader perspective in which the relative contributions inflammation, axonal loss, and neuronal degeneration are weighed in the balance. Accordingly, there is a need for noninvasive methods that capture the interactions between different pathogenic mechanisms that contribute to progression and disability in MS. As a putative model of MS, the afferent visual pathway (AVP) offers several potential advantages.

### 3.1. Localizing a Sentinel Lesion

Eighty percent of MS patients present with an acute clinical episode affecting one or several neurological sites, which is known as the clinically isolated syndrome (CIS) [1, 23]. As an inflammatory lesion of the optic nerve, optic neuritis (ON) is the best characterized CIS, representing the initial clinical event for 21% of MS patients [23]. Much of what we have come to understand about ON has been derived from the long-term followup of the Optic Neuritis Treatment Trial (ONTT) [24–27], which demonstrated that most ON patients are young (mean age 32 years) Caucasian (85%) women (77%). Ninety-two percent of ONTT patients reported pain at the onset of vision loss [24–26], which is often characterized as an “ache” made worse with eye movement. Vision loss is generally subacute, progressing over hours to days. The severity of vision loss in ON may range from mild to no light perception initially, and dyschromatopsia or decreased color vision is common. In cases of retrobulbar ON, the fundus examination is initially normal, whereas patients with anterior ON or “papillitis” may manifest optic disc swelling acutely. The initial period of visual recovery occurs with a period of weeks, and further improvement in vision is seen up to a year after the acute episode [24–27]. With ON as its inflammatory relapse prototype, the AVP model provides objective evidence of a sentinel lesion, which can be precisely localized in the CNS.

### 3.2. Defining Time of Onset

As foveating animals, humans are “hard-wired” to seek high resolution images in the world around them. Thirty-eight percent fibers carrying information to and from the brain are contained within the optic nerves (an estimated sum of 2.5 million axons) [28], and if the rods and cones within the retina are considered separately, the total of sensory units that forward afferent input into the brain is increased by a factor of eighty [28]! In light of the highly specialized nature of the AVP, any perturbation in the system that interferes with visual perception, particularly central vision, will be noticed and often reported by affected individuals. Moreover, the course of functional recovery after ON can be monitored from a precise point of onset in the AVP model of MS.

### 3.3. The Link between Structural Integrity and Functional Recovery

The AVP is a functionally eloquent CNS system, and deficits can be captured with highly reproducible measures including high- and low-contrast visual acuity, automated perimetry, and color vision testing. Moreover, paraclinical tests including optical coherence tomography, visual evoked potentials, and motion perception techniques can be used to explore both the functional and structural integrity of the AVP. In this manner, a structural-functional paradigm can be devised to elucidate the temporal evolution and relative contributions of inflammation, axonal loss, neuronal damage, and cortical compensation in the AVP model of MS.

### 3.4. The Back of the Eye Is the Front of the Brain

Previous pathological studies have shown that tissue-specific injury in the AVP mirrors global CNS effects in MS patients [15]. With an acute ON event, cytokine release induces transient conduction block, probably caused by damage induced by nitric oxide [29]. When myelination and axonal integrity are intact, recovery ensues with the removal of inflammatory mediators [29]. During recovery from ON, remyelination improves saltatory conduction through sodium channels, which are distributed along the demyelinated optic nerve segment [29]. Cortical plasticity is also believed to play a role in optimizing function in the more chronic phases of recovery [29], albeit the timeline and mechanisms involved therein are not well understood. The AVP model can be used to identify tissue-specific and system-based factors that govern injury and repair in MS.

## 4. The Afferent Visual Pathway: Clinicoanatomic Correlations

*“Form and function should be one, joined in a spiritual union [30].” *

**Figure 1:**
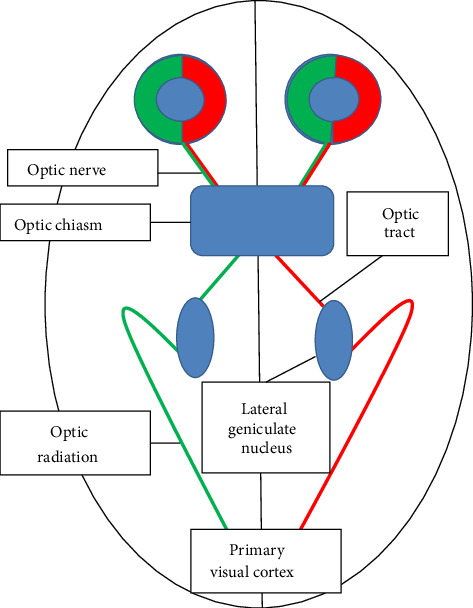
A schematic diagram of the afferent visual pathway.

Frank Lloyd Wright was referring to architecture with this version of his iconic comment, but these principles apply equally well to the visual system, in which anatomical integrity and clinical function are tightly linked, allowing precise topographic localization of pathological lesions. The AVP originates in the retina, where the first order neuron begins with the bipolar cells [28]. The second order neuron extends from the retinal ganglion cells (RGCs) to the lateral geniculate nucleus (LGN) in the thalamus, and the third order neuron leaves the LGN en route for the visual cortex [28]. To fully appreciate how form relates to function in the AVP, one must first consider its integral parts including the retina, optic nerves, optic chiasm and tracts, LGN, optic radiations, and the visual cortex (Figure 1).

**Figure 2:**
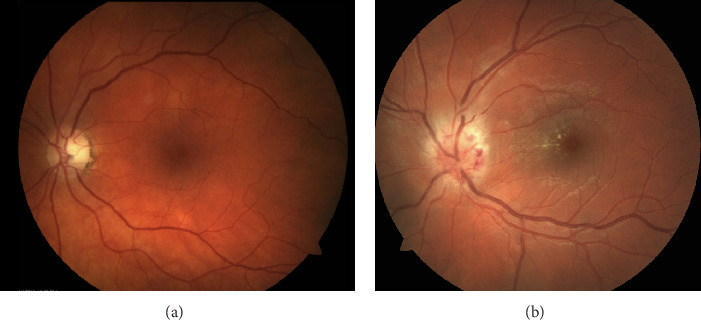
(a) A fundus view of the optic nerve and macula. (b) A fundus view of a swollen optic nerve with associated macular edema in a patient with neuroretinitis.

*The Retina *extends anteroposteriorly from the ora serrata to the optic disc [28]. When visualized with ophthalmoscopy, the macula appears yellow relative to the surrounding retina and is located temporal to the optic disc (Figures 2(a) and 2(b)) [31]. At the center of the macula lies the foveola, which is devoid of vessels and other neuronal elements, other than the tightly packed cones [31]. Visual discrimination is greatest in this region of the retina, as light reaches the photoreceptors by passing through the thinnest regions of the retina. On a microscopic level, the retina consists of several major layers through which light is transduced [32, 33]. Photoreceptors connect to bipolar cells, which in turn relay messages to RGCs [32, 33]. In concert, horizontal and amacrine cells form lateral connections between elements of these layers. Bipolar cells then provide inputs to RGCs via direct excitatory glutamatergic synapses or indirect, inhibitory GABAergic connections [32]. The process of converting light into sight begins in the outer retina, which is comprised of photoreceptor cells (rods and cones). Photoreceptors transmit signals which are processed in the RGC layer. Retinal ganglion cells differ in type such that large magnocellular (M) cells make up approximately 5–10% of RGCs and are concerned with “where” visual targets are in space [31]. These cells have large receptive fields and propagate action potentials quickly [31, 32]. The M cells are color “ignorant” and have high-contrast sensitivity, low spatial resolution, and high temporal resolution [31, 32]. In contrast, small parvocellular (P) cells represent approximately 90% of the RGC population. The P cells are predominant in the macula and are concerned with “what” is being seen [31, 32]. Specifically, P cells facilitate low-contrast sensitivity and high spatial resolution vision [31, 32]. Certain diseases are believed to preferentially affect M cells or P cells. In Alzheimer`s disease, for example, there is a predominant loss of M cells in the retina, with consequent problems of determining depth and motion perception. In contrast, ON has traditionally been viewed as a disorder of P cells more so than M cells, because conventional testing has shown these patients to have central vision loss, dyschromatopsia, and persistent contrast sensitivity abnormalities [31]. There is emerging evidence to suggest that neuronal damage in both the outer and inner layers of the retina may occur as a primary process in MS.

### 4.1. The Retinal Nerve Fiber Layer

**Figure 3:**
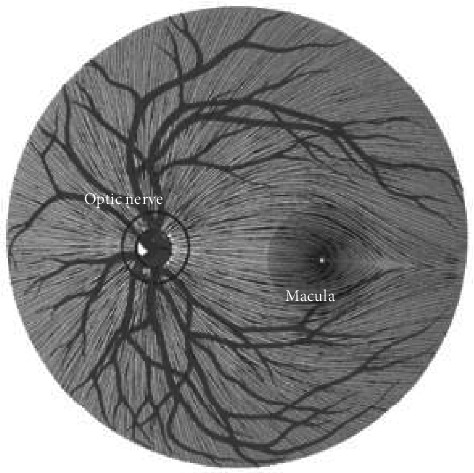
The topographic arrangement of the retinal nerve fiber layer indicating the optic nerve and macula. Nasal RNFL fibers radiate into the optic disc like the spokes of a wheel, whereas the large temporal fibers sweep superiorly and inferiorly around the central area of the retina from the periphery. Small densely packed fibers radiate directly to the mid-temporal optic disc edge from the fovea, making up the papillomacular bundle (image provided by Dr. Kathleen Digre).

Light information is transferred along the axons of the RGCs which reside in a transparent region of the inner retina, referred to as the retinal nerve fiber layer (RNFL) [32]. The RNFL has an intricate topographic arrangement, which readily allows identification of visual field defects that arise from an optic nerve injury (Figure 3) [32]. The RNFL lacks myelin, and defects therein are interpreted to represent damage to the RGC axonal integrity [34]. Prior to the modern ocular imaging era, RNFL defects were visualized with direct ophthalmoscopy and red-free photography. With the advent of optical coherence tomography, it is now possible to quantify changes in RNFL thickness as a surrogate marker of axonal integrity in the AVP, which typically progress for up to a year after acute ON [35, 36].

### 4.2. Optic Nerves, Chiasm, and Tracts

The axons of the RFNL converge on the optic disc to exit the back of the eye through the optic nerve. The optic nerve acquires myelin behind the eye at the lamina cribrosa and is comprised of approximately 1.2 million RGC axons contained within the RNFL [32]. The intraocular segment of the optic nerve head (the optic disc) is located 3-4 mm nasal to the fovea and is 1 mm thick. Within the optic nerve, a strict topographical arrangement is maintained as visual fibers originating in the RGC make their way to the LGN: superior fibers run superiorly, inferior fibers reside below, and those from the temporal and nasal retina run in the corresponding parts of the nerve [31]. The optic nerves consist of myelinated nerve fibers similar to those forming white matter tracts elsewhere in the brain, which makes them vulnerable to inflammatory demyelinating injury in MS.

### 4.3. Optic Chiasm

Both optic nerves converge in the anterior compartment of the skull to form the optic chiasm, which lies over the sella turcica [31, 32]. Within the optic chiasm, approximately 50% of the fibers originating from the RGCs of the nasal retina cross to reach the contralateral optic tract [31, 32]. Uncrossed fibers, originating from the temporal retina, maintain their dorsal and central position in the chiasm [31, 32]. This arrangement allows for each side of the brain to see the opposite side of the world (Figure 1). 

### 4.4. Optic Tract

The postchiasmatic portion of the AVP is comprised of the left and right optic tracts [31]. Within each optic tract, axons course from the ipsilateral temporal retina and the contralateral nasal retina. Tract lesions which disrupt the RGC axons destined for the LGN cause retrograde degeneration, which can be viewed as “band atrophy” of the optic nerve.

### 4.5. The Lateral Geniculate Body

The LGN provides a relay station for the RGC axons and exerts dynamic control upon the amount and nature of information that is transmitted to the visual cortex [31, 32]. Neurons from the LGN project by way of the optic radiations to the calcarine cortex of the occipital lobe. In addition to retinal afferents, the LGN receives modulating connections from the thalamic reticular nucleus and layer 6 of the visual cortex. It thus provides a bottleneck to information flow, filtering visual information for relevance to the present behavioural state [32].

### 4.6. The Optic Radiations

The third order neurons of the AVP extend from the LGN to the visual calcarine cortex located in the occipital lobe. These neurons are grouped into the temporal radiations which take an anterior course through the temporal pole (Meyer's loop) before turning in a posterior direction, and the parietal radiations [32]. A retinotopic arrangement is maintained at the level of the cortex, such that temporal radiations represent the contralateral superior visual field and parietal radiations represent the contralateral inferior field [31, 32]. 

### 4.7. Visual Cortex

Cortical area 17 is located along the superior and inferior banks of the calcarine fissure in the medial aspect of the occipital lobe [31, 32]. The visual cortex receives axons from the neurons of the LGN projected via the optic radiations and represents the first link in the cortical processing of visual information. Each occipital lobe receives projections from the nasal half of the opposite eye and from the temporal half of the ipsilateral retina [31]. The superior and inferior retinal projections extend to the superior and inferior banks of the calcarine fissure, respectively [31]. The macular area is represented in the posterior pole of the calcarine cortex, whereas the more peripheral retina is more represented more anteriorly [30]. There is a 300–400-fold increase in the number of neurons in the primary visual cortex compared to the retina or LGN, with with 350 million neurons housed at a density double that of other cortical areas [32]. The macula representation is highly magnified in the visual cortex retinotopic map such that connections from 1 mm^2^ of the retina representing the central 10 degrees of visual field make up 60% of the striate cortex! [32]. The correlate of this cortical magnification is high central acuity. The peripheral parts of the visual field are served by more anterior portions of the striate cortex [31].

In addition to its topographical organization, the visual cortex is divided in a columnar network [32]. The cells contained in each column have the same orientation as their receptive fields. There are rich intercommunications between cortical cells in a vertical direction so that each column of cells can be viewed as a functional unit [32]. Monocular inputs to the cortex are arranged in ocular dominance columns. Because our two eyes have different views of visual space, there is a slight displacement of their respective retinal images [32]. At the binocular fixation point, an image is projected onto anatomically corresponding retinal locations [32]. Objects in front of or behind the binocular fixation point give rise to noncorresponding images [32]. This retinal disparity is the basis of cortically perceived stereoscopic depth [32]. The synergistic effect is a rich 3D perception.

## 5. Using the Afferent Visual Pathway Model to Explore Pathogenic Mechanisms of Disease in Multiple Sclerosis

Within the AVP, there are the tissue-specific substrates that sustain visual function, including the retinal elements; the myelinated components of the optic nerve, chiasm, tracts, and radiations; and the higher order cortical processing centers. Given the capacity for CNS plasticity and functional adaptation, clinical recovery from an AVP lesion might not simply result from structural repair within the primary lesion alone. Restoration of function may be influenced to some extent by “cortical reserve,” which may vary with the age and stage of MS. Furthermore, chronic inflammation and the capacity for remyelination may also play a role in recovery. Tests of structure and function capture changes in the integrity of the AVP.

### 5.1. Testing Functional Integrity in the Afferent Visual Pathway Model

#### 5.1.1. High-Contrast Visual Acuity (HCVA)

High-contrast visual activity refers to spatial resolving ability of the eye [37] and has long been the mainstay of standard visual testing. Theoretically, HCVA is a measure of macular function, a presumed parvocellular mediated pathway, but in reality it reflects the structural-functional integrity of the entire AVP. High-contrast visual acuity has long been considered “gold standard” for primary outcomes of clinical trials in ophthalmology, yet it is a relatively crude measure of afferent visual function in MS [38]. Many patients will report significant visual dysfunction even with Snellen visual acuity equivalents of 20/20 vision [38]. For all of its limitations, HCVA testing has some predictive value in forecasting functional recovery after ON. Kupersmith and colleagues [39] used the ONTT database to evaluate various cutpoints for baseline and 1-month vision levels that predicted abnormal 6-month vision. Failure to reach a 1-month HCVA cut-off of 20/50 correlated with having moderate-to-severe loss in this domain of function after six months [39]. Thus, HCVA measures can be used to identify patients with potentially poor visual recovery after ON in future neuroprotective studies employing the AVP model of MS. 

**Figure 4:**
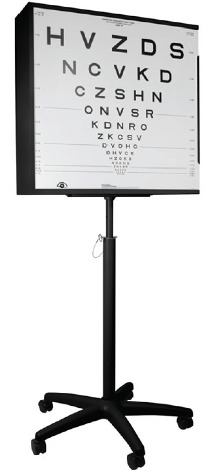
Early Treatment Diabetic Retinopathy Study (ETDRS) chart (image provided by Dr. Laura Balcer).

The most common HCVA charts employed in routine clinical practice and clinical research studies include the Snellen and Early Treatment Diabetic Retinopathy Study (ETDRS) charts (Figure 4) [37]. The Snellen chart has letters of different sizes arranged from largest at the top to smallest at the bottom, which are read, at a distance of 6 meters (20 feet) [37]. Snellen visual acuity is usually expressed as a fraction with the numerator equal to the distance from the chart and the denominator being the size of the smallest line that can be read. The reciprocal of the fraction equals the angle, in min of arc, that the stroke of the letter subtends on the patient's eye and is called the minimum angle of resolution (MAR) [37, 40]. There are numerous disadvantages of Snellen charts, which compromise the reliability of this testing modality in the clinical and research arenas [37]. Consequently, the ETDRS chart has become the “gold standard” HCVA test for most current clinical trials. The ETDRS charts are superior to Snellen charts because interpatient differences are more accurately measured and longitudinal follow-up measurements have more consistent precision, regardless of whether the patients have high or low levels of visual acuity [37]. The ETDRS method allows visual acuity to be converted to logMAR, which converts the geometric sequence of a traditional chart to a linear scale [37, 40]. In logMAR notation, lower scores correspond to better vision, and as acuity becomes worse, the value of the logMAR increases.

#### 5.1.2. Low-Contrast Letter Acuity and Contrast Sensitivity (LCLA)

**Figure 5:**
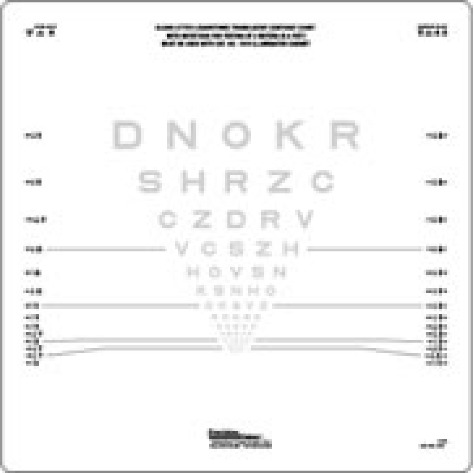
Low-contrast letter acuity chart (image provided by Dr. Laura Balcer).

While acuity is a visual measure determined under optimal circumstances of high contrast and high luminance, the real visual world is one of varying spatial and temporal frequencies, contrast, color, luminance, and glare [38, 41–44]. In many respects, contrast acuity and low-contrast letter acuity (LCLA) testing (Figure 5) attempt to capture the many shades of grey that color our day to day lives. Low-contrast testing identifies the minimum size at which letters of a particular contrast level can be perceived [44]. Low-contrast letter acuity charts have a standardized format based on the ETDRS visual acuity charts and different contrast levels (100%, 5%, 2.5%, 1.25%) [41–43]. Visual improvement and loss by the low-contrast acuity chart has been defined as a 7-letter change in score [42, 43]. As defined, these LCLA deficits have been shown to correlate with quality of life measures and neurological disability scores in MS patients.

Balcer and colleagues [41–45] have spearheaded our understanding of how LCLA can be used to measure functional integrity in the AVP. In a substudy of the International Multiple Sclerosis Secondary Progressive Avonex Controlled Trial (IMPACT), mean letter scores were generally lower for patients with MS compared with healthy volunteers for all four contrast levels studied, with the greatest difference noted at the lowest contrast level [42, 43]. The discrepancies were observed despite similar median visual acuities based on the Snellen visual acuity equivalent (100% contrast level). Similarly, in patients from the Multiple Sclerosis Vision Prospective (MVP) cohort study, MS patients and healthy volunteers had similar letter scores at 100% contrast, whereas the former had lower letter acuity scores for Sloan charts with contrast levels of 5%, 2.5%, and 1.25% [42, 43]. 

Contrast can be defined as a measure of the difference between the luminance of the object and its surroundings. Contrast sensitivity function is conventionally measured by finding the threshold contrast of sine wave gratings of varying spatial frequencies (sizes) [44]. Visual acuity is almost constant for all high-contrast levels, whereas at lower contrasts, acuity becomes strongly dependent on contrast changes. Fisher and colleagues [38] showed that contrast sensitivity scores were worse among eyes of MS patients compared with disease-free controls. Moreover, the ON eyes of MS patients had significantly worse contrast sensitivity scores than MS eyes without a history of ON [38]. Hence, together LCLA and contrast sensitivity testing are more sensitive in detecting visual deficits in spatial frequency than HCLA testing in MS, and inclusion of these functional outcomes in the AVP model will better capture deficits that impact quality of life and day- to- day function in these patients.

#### 5.1.3. Binocular Summation of Visual Acuity

Binocular viewing is superior to monocular vision when it comes to threshold tasks such as contrast detection, due to a phenomenon called binocular summation [45]. In contrast, patients with binocular inhibition have worse binocular vision as compared to the monocular view they get with their better seeing eye [43, 45]. In a recent study of 1831 individuals, prevalence rates of binocular summation and inhibition were 21% and 2%, respectively [46]. Compared with participants less than 65 years old or those with equivalent interocular visual acuity, older participants (≥65 years) and those with interocular differences in visual acuity were more likely to demonstrate binocular inhibition [46]. It was noteworthy that participants with binocular inhibition had greater self-reported problems with driving activities [46]. The phenomena of binocular summation and inhibition are not well understood but may relate to neural interactions of input from both eyes within the post-geniculate visual pathway [45]. Given that binocular summation is likely mediated at the cortical level, MS patients may experience functional limitations due to interruptions in normal cortical signaling [45]. A study of 1,007 patients with MS and 324 disease-free controls showed that binocular summation was substantial for low-contrast acuity at the 2.5% and 1.25% levels [45]. With HCVA, only 3.0%–3.4% of patients showed similar degrees of summation [42, 45]. Increasing age, greater interocular differences in acuity, and history of ON were associated with lower magnitudes of binocular summation and worse binocular inhibition [45]. Hence, binocular summation may provide us insights about the day-to-day visual challenges of MS patients that are poorly captured with standard high-visual acuity testing. Further exploration into the phenomena of binocular inhibition and summation could help us better understand the role of cortical adapation in visual recovery after a CNS insult.

#### 5.1.4. Critical Flicker Fusion Frequency (CFFF)

Critical flicker fusion frequency is defined as the lowest frequency at which a flickering light is perceived to be nonflickering or “steady” [47–50]. As a test of visual function, CFFF is a rapid and simple technique that can provide information about the responsiveness of the visual system by defining the upper limits of temporal resolution. Studies have shown that CFFF perception improves with increased target luminance, target size, and retinal eccentricity, whereas decreased CFFF perception occurs with age [47–50]. Testing CFFF is important not only in assessing the integrity of the retina but also reflects the capabilities and limitations of neural processing with respect to the speed and transmission of the neural response. Previous reports have shown that the magnocellular system is primarily involved in the processing of rapid flicker and motion and that CFFF is affected by optic nerve damage, especially demyelination [50]. In a prior study of 25 ON patients, CFFF results were 100% abnormal initially and gradually improved over time. However, even in recovery CFFF abnormalities were noted in 37% of ON eyes [48]. There were also CFFF abnormalities in the fellow (non-ON) eyes of patients with unilateral ON. There is therefore evidence to suggest that CFFF could potentially be compared with other measures of spatial and temporal frequency to interrogate the integrity of myelination and neuroaxonal integrity in the AVP model.

#### 5.1.5. Color Vision

In ON patients, color vision is often decreased acutely and improves over time. Various techniques have been used effectively to capture color vision deficits in MS patients [51–54]. Hardy-Rand-Rittler (HRR) pseudoisochromatic color plates have demonstrated an advantage over the Ishihara method, because the former are more sensitive to red-green and blue-yellow deficits caused by neuroophthalmic disorders [51]. Recently Villoslada [52] studied 213 MS patients and 47 healthy controls to determine the relationship between HCVA, LCLA, color vision (HRR plates and Lanthony D15 tests), and optical coherence tomography (OCT). Multiple sclerosis patients showed HCVA and LCVA deficits but exhibited even more profound abnormalities in color discrimination relative to controls [52]. Moura and colleagues [54] assessed chromatic discrimination in 35 MS patients (with and without ON) and 74 age-matched controls using the Cambridge color test (CCT) to determine the magnitude and chromatic axes of any color vision loss in both patient groups and to evaluate age-related changes in chromatic discrimination in both patient groups compared to normal control subjects. Color thresholds for both ON eyes and non-ON eyes in MS patients were significantly higher than control eyes along the protan and tritan axes [54]. In addition, MS patients manifested progressive color discrimination impairment with age (along the deutan and tritan axes) that was almost two times faster than controls, even in the absence of ON [54].

#### 5.1.6. Perimetry

**Figure 6:**
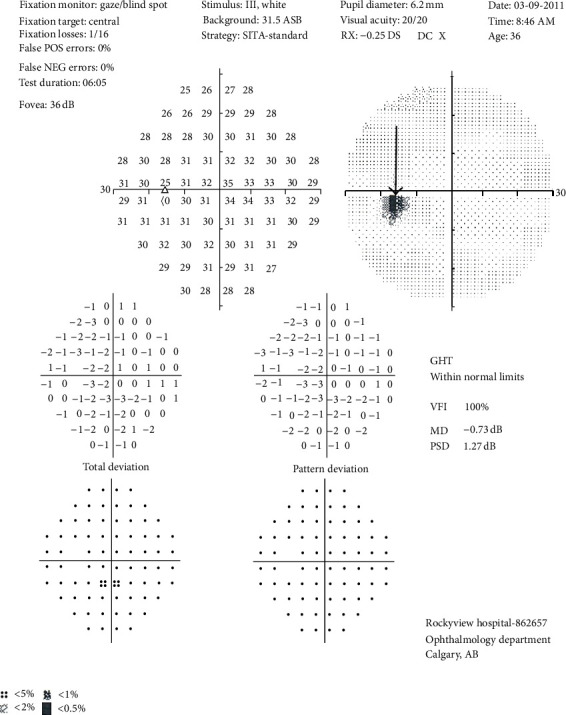
Humphrey perimetry 30-2 threshold testing of the left eye showing the normal physiological blind spot (arrow).

Visual field testing has been described as the cornerstone of the sensory visual examination and provides invaluable information about the integrity of AVP function from the retina to the visual cortex  [55]. Perimetry has evolved through stages since original confrontation-based techniques to allow quantification and statistical analysis in its currently used computerized forms (Figure 6). This provides critical information about visual function including both central and peripheral channels, and many of the perimetry machines in common use today are readily available in most cities around the developed world allowing for easier comparisons across offices over time. In the 15-year followup from the ONTT, Keltner and colleagues [56] defined visual field characteristics and classifications for the entire cohort, from baseline through 15 years. At presentation, 100% of the visual fields from the ON eyes and 75% of the visual fields from the fellow (non-ON) eyes in patients were abnormal. After year one, the frequencies of abnormal and normal visual fields for the affected eye were evenly distributed at approximately 50% each, whereas the abnormal visual field frequency in the fellow eye ranged between 34% and 40% [56]. Diffuse and central visual field deficits were more prominent in ON eyes than fellow eyes at baseline, and nerve fiber bundle defects (partial arcuate, paracentral, and arcuate) were the most prominent localized abnormalities in the affected and fellow eyes during the study [56].

For all of the established advantages of automated perimetry, there are potential pitfalls in applying these techniques to a patient population that is subject to fatigue-related visual dysfunction. Wall and colleagues [57] followed 17 patients with ON and 10 healthy control subjects with repeat intraday and interday automated perimetry testing (five Humphrey 30-2 tests were administered during a 7-hour period on the same day and at the same period on 5 separate days). Optic neuritis patients demonstrated variations in visual field sensitivity that were outside the entire range of variability for normal controls [57]. These variations occurred for multiple tests performed on the same day, at specific times, and for tests performed at specific times on different days [57]. Thus, when using automated perimetry, distinguishing true change from variability remains a challenge. 

#### 5.1.7. Motion Perception Testing

Previously, Barton and Rizzo [50] used motion perception techniques to study 13 patients with optic neuropathies and 19 healthy control subjects. Motion perception and CFFF testing results were “double-dissociated” meaning that eyes could have abnormalities in one or the other, without necessarily having correlating abnormalities in both. This finding refuted the notion that both motion perception and CFFF were mediated exclusively by a common magnocellular pathway [50]. The hypothesis that ON can have prolonged effects on visual motion processing, which may persist after there has been an objective return to normal “form” perception, was further explored by Raz and colleagues in a series of elegant papers [58, 59]. They prospectively followed 21 ON patients over one year with tests of spatial and dynamic visual functions including visual acuity, perimetry, contrast sensitivity, color vision, visual evoked potentials, and OCT testing [58]. In addition the authors developed a novel set of motion perceptual tasks to test dynamic visual deficits as part of their study protocol [58]. In ON eyes, visual acuity, visual field, and colour vision deficits were apparent in the acute phase and subsequently improved after one month [58]. Contrast sensitivity deficits persisted somewhat longer, improving 4 months after symptom onset [58]. As compared to tests of spatial visual function, motion (temporal) perception was impaired during the full follow-up period of one year [58]. Thus motion perception testing revealed the most significant and prolonged impairment after ON, and motion perception problems were independent of contrast sensitivity levels [58]. 

The investigators then endeavored to identify mechanisms underpinning the sustained deficit in dynamic visual function following ON [59]. They hypothesized that motion perception may be more vulnerable to slowed conduction in the optic nerve, which could be measured with visual evoked potential (VEP) testing [59]. To explore this theory further they performed motion perception and VEP testing at presentation, 1 month, 4 months, and 12 months after ON [59]. The VEP amplitudes in ON eyes were significantly reduced compared to fellow eyes in the acute phase, but these differences resolved in later phases of recovery [59]. In keeping with the aforementioned findings of Kupersmith and colleagues [39] visual performance 1 month after ON was highly predictive of visual recovery, as determined with visual acuity, contrast sensitivity, and motion perception testing [59]. Intact VEP amplitudes were associated with recovered visual acuity and contrast sensitivity after ON, suggesting that these visual functions depend on a sufficient amount of visual information reaching the cortex [59]. In contrast, motion perception was impaired even in patients with intact VEP amplitudes, indicating that an intact amount of visual projection alone does not impact dynamic visual function [59]. Instead, while the magnitude of contrast sensitivity improvement related to the extent of VEP amplitude restoration, the magnitude of motion perception improvement depended on the extent of VEP latency reduction post-ON [59]. From these findings, it was inferred that there is a need for rapid transmission of visual input to perceive motion. Moreover, motion perception testing in concert with VEP may serve to assess the impact of demyelination/remyelination in the AVP model of MS.

#### 5.1.8. Visual Evoked Potential Testing

The VEP is a response of the brain to repeated visual stimulation and has traditionally been recorded when visual field is stimulated with a single checkerboard pattern [60–65]. The VEP is known to be generated at the level of striate cortex by the combined activity of postsynaptic potentials [60–65]. The magnitude of the VEP reflects the number of functional afferent fibers reaching the striate cortex [60–64]. In ON patients, diminished VEP amplitudes indicate inflammation-induced conduction block, axonal atrophy, or a combination of both [29]. Subsequently, increased VEP amplitudes which occur after ON are a consequence of resumed conduction in previously blocked fibers due to resolution of inflammation and edema or possibly expansion of synaptic activity along the visual pathway up to the level of V1 [29]. Delayed VEP conduction is recognized as one of the earliest features of acute ON, with the subsequent shortening of latency thought to represent the process of remyelination.

**Figure 7:**
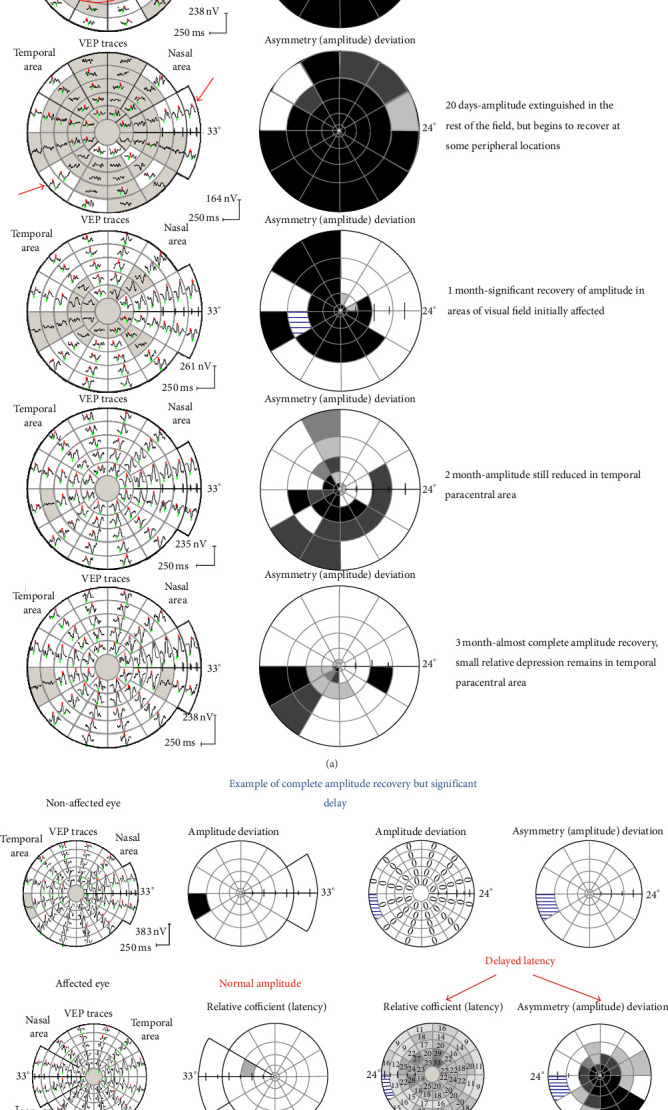
(a) Multifocal VEP amplitude chages after acute optic neuritis. (b) Multifocal VEP showing an example of complete amplitude recovery but significant latency delay (Image provided by Dr. Alexandr Klistorner).

Because it is a summation of a large number of neuronal elements, the full-field VEP is greatly dominated by the macular region due to its cortical overrepresentation [60, 61, 63, 64]. Moreover, the waveform of the full-field VEP is prone to cancellation and distortion, which sometimes leads to apparent, rather than real, latency delay [60, 61, 63, 64]. In contrast, the multifocal VEP (mfVEP) allows stimulation of small areas of the visual field [60, 62–64]. The result is a detailed topographical assessment of small groups of axons within the optic nerve and visual cortex, which is resistant to waveform distortion [60, 62–64] (Figure 7). In a recent study, Klistorner et al used mfVEP and OCT testing to prospectively study 25 subjects with acute unilateral ON. While mfVEP amplitude asymmetry at baseline varied significantly among the patients, it was, on average, very high, indicating considerable reduction of amplitude in ON eyes. The intereye asymmetry in mfVEP amplitude decreased over time indicating continuous functional recovery [60]. There was an insignificant negative correlation between the inter-eye asymmetry of OCT-measured RNFL thickness and that of mfVEP amplitude at one month. This was consistent with vasogenic edema in the acute phase, causing an increase in RNFL thickness, with a corresponding reduction in mfVEP amplitude [60]. Over the course of recovery, the correlation became more robust, suggesting the diminishing role of optic nerve edema in measured RNFL thickness and unmasking the association between RNFL atrophy and low mfVEP amplitude [60]. The potential correlation between OCT-measured RNFL values and mfVEP measures of anterior visual pathway damage was demonstrated by the same group, who evaluated 32 patients with unilateral ON and 25 control subjects [63]. The mean RNFL thickness in ON eyes (85 *μ*m) was reduced by 19% compared with control eyes (104 *μ*m), and there was a 40% reduction in the amplitude of the mfVEP in ON eyes relative to control eyes [63]. In addition to demonstrating the utility of mfVEP in tracking optic nerve injury in ON patients, this study further confirmed the significant correlations between structural and functional measures of optic nerve integrity and showed that demyelination contributes to axonal loss [63]. It may therefore be feasible to pair mfVEP and OCT testing to capture the synergistic effects of acute demyelination and axonal loss over time in ON/MS patients. Furthermore, the putative relationship between the VEP latency and axonal loss encourages the notion that therapeutic interventions aimed at reducing the effects of demyelination or enhancing remyelination may be trialed in the AVP model.

#### 5.1.9. Electroretinography

The electroretinogram (ERG) provides an objective, quantitative measure of functional integrity in the photoreceptors (rod and cones) and ganglion cells in the retina. Electrodes are placed on the cornea or adjacent to the orbit to monitor changes in the electrical potential of the eye in response to specific stimuli [66]. The full-field ERG is the most common form of ERG testing, and prior reports employing this technology have shown that outer retinal dysfunction occurs in MS [67, 68]. Forooghian et al. [68] studied 34 MS patients and 37 healthy control subjects with standard ERG testing and a novel bright flash ERG protocol technique to detect evidence of rod photoreceptor function [68]. Patient and control sera were analyzed for the presence of antiretinal antibodies in this study, using Western blot techniques [68]. They observed significant differences between MS patients and controls in four ERG parameters: in the MS group, implicit times of the rod-cone b-wave response, cone b-wave response, and rod photoreceptor response were increased, whereas the amplitudes of the photopic oscillatory potentials were reduced in the MS group relative to control subjects [68]. Interestingly, MS patients with the highest titres of retinal autoantibodies had delayed rod-cone b-wave implicit times and diminished photopic oscillatory potential amplitudes [68]. Anti-retinal antibody reactivity against retinal antigens including arrestin and *α*-enolase has previously been described in MS [69] suggesting that retinal autoimmunity may be the basis of retinal and specifically rod photoreceptor dysfunction in the disease [69]. Thus ERG testing could potentially be used in the AVP model to explore the role of autoimmune mechanisms underlying outer retinal dysfunction in MS patients. 

While the full-field ERG enables the assessment of general retinal function, it cannot provide specific information about individual sectors of the retina, which is needed in the setting of multifocal or regional disease [70]. In contrast, the multifocal ERG (mfERG) measures the response in each of a large number of small sectors, thus providing a map that allows the clinician to locate specific areas of malfunction in the retina [70]. The mfERG is particularly valuable in cases in which the fundus appears normal, and attempts are being made to localize a disease process to the outer retina, retinal ganglion cells, or optic nerve [70]. Recently, mERG testing was used to confirm the presence of retinal abnormalities in MS patients, described as manifesting the predominantly macular thinning phenotype (MTP) [16]. This phenotype was so-named to describe patients with deeper disruption of retinal architecture than would be expected due to retrograde degeneration from either typical clinical or subclinical optic neuropathy [16]. Functional corroboration of retinal dysfunction was provided through mfERG testing which demonstrated diffuse abnormalities, indicating that MS may target the anterior visual pathway at multiple sites, including the optic nerve (with subsequent axonal and neuronal degeneration in the retina) and the retina itself (involving discrete pools of neurons). 

## 6. Testing Structural Integrity in the Afferent Visual Pathway Model

### 6.1. Optical Coherence Tomography

Since the invention of the ophthalmoscope, the structural consequences of optic nerve injury have been visualized acutely as optic disc edema, followed by disc pallor and corresponding defects in the RNFL [34, 42, 71]. The RNFL represents a unique CNS structure, because it lacks myelin, and changes therein have been interpreted to represent axonal loss caused by anterograde retinal damage or retrograde degeneration from a retrobulbar optic nerve injury [71]. Experimental models have shown that in eyes with total optic nerve transection, the disappearance of normal RNFL striations begins at one month and is completed by two months [71, 72]. Yet, other reports have indicated that retrograde degeneration may take as long as 6 months to fully develop [71, 73]. Given that the back of the eye represents the front of the brain, it is intuitive that structural damage in the retina occurs the setting of MS. In 1974, Frisen and Hoyt [34] reported RNFL defects as evidence of axonal attrition in MS patients; recent work by Green et al. [15] has provided postmortem evidence for retinal atrophy and inflammation in MS patients, even in late stages of the disease.

**Figure 8:**
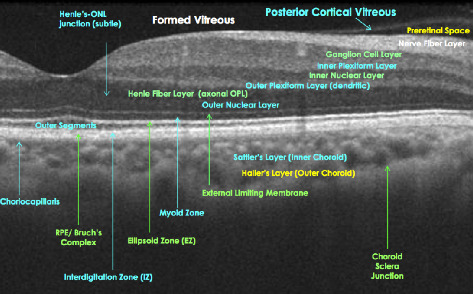
OCT line scan of the macula with consensus layer nomenclature labeling of layers and zones (SPECTRALIS^®^ image courtesy of Heidelberg Engineering).

In the modern ocular imaging era, OCT has allowed us to acquire high-resolution, noninvasive imaging of retinal architecture (Figure 8). Changes in peripapillary RNFL thickness as measured by OCT have been interpreted to represent axonal damage [10, 34, 36, 38, 52, 53, 58, 59, 63, 64, 71, 74–83] whereas loss of macular volume [53, 74] and RGC layer thinning [84, 85] have been viewed as evidence of neuronal pathology in MS which may occur as a primary or secondary phenomenon in the disease. Previous OCT studies have shown that at the time of an acute inflammatory ON event, when vision loss is at its nadir, patients manifest RNFL measurements that are either comparable to or increased in their affected eye (ON eye) relative to their unaffected eyes [10]. Correspondingly, the optic nerve in the ON eye tends to be mildly edematous or hyperemic secondary to axoplasmic flow stasis [10]. After two to three months, optic disc pallor and RNFL thinning evolve, with earliest signs of significant RNFL atrophy manifesting in the temporal RNFL region [10]. In ON eyes, RNFL values continue to decrease for six to twelve months after symptom onset, plateauing thereafter [10]. A year after an isolated ON event, peripapillary RNFL measurements are reduced by approximately 20% relative to the fellow eye [10]. In a recent meta-analysis of time domain OCT studies (14 studies (2,063 eyes)) RNFL values were reduced from 5 to 40 *μ*m (averaging 10 to 20 *μ*m) in ON eyes of MS patients [83]. Furthermore, comparing ON eyes in MS patients with the eyes of healthy controls showed an estimated average RNFL loss of 20 *μ*m [83]. Lower RNFL values have been shown to correlate with reduced visual acuity [53, 71], visual field mean sensitivity [53, 71], and color vision testing scores [53]. For patients selected without recruitment bias, an OCT “cut-off” of 75 *μ*m has been shown to represent a threshold of RNFL integrity that can predict the extent of visual recovery after ON [71]. 

For any given MS patient, it is difficult to know whether retinal damage arises as a primary “neuronopathy” or whether damage to RGCs and deeper neuronal elements in the inner nuclear layer occur as a dying back consequence of retrograde axonal degeneration from a retrobulbar optic nerve injury. Multiple sclerosis patients manifest retinal inflammatory changes including periphlebitis and pars planitis, in a region of the AVP that lacks myelin. This finding challenges the premise that myelin debris is the only antigenic trigger in this disease [84]. Previous studies assessing retinal pathology in MS have demonstrated atrophy of the RNFL and RGC layers [15, 85]. Green and colleagues [15] observed shrunken neurons, dropout of RGCs (in 79% of MS eyes), and inner nuclear layer atrophy (in 40% of MS eyes) [15]. The finding of inner nuclear layer atrophy indicated that neuronal pathology is not restricted to the RGC layer in the eyes of MS patients and that retinal injury is more widespread than previously appreciated [15]. In this study, the severity of retinal atrophy was significantly associated with postmortem brain weight, and there was an association with disease duration, suggesting that the observed retina pathology may reflect global changes occurring in the CNS over time [15]. With the exception of demyelination, virtually all manifestations of brain tissue injury in MS can be found in the retina. Thus, using OCT in the AVP model may help us decipher different types of retinal pathology and enhance our understanding of the factors that drive both inflammation and tissue atrophy in MS.

At this point, it is not known whether structural disruption occurs in retinal layers deeper than the inner nuclear layer in MS. If MS affects the outer retina directly, it could indicate that primary retinal neuronal pathology is pathogenic feature of the disease. Arguably, RGC layer and inner nuclear layer damage may occur as a consequence of a direct immune-mediated process; or in keeping with the tenants of the Inside Out model, RGCs and deeper neuronal structures may be targeted by a common neurodegenerative process. Alternatively, as has been proposed by Green et al. [15] loss of retinal neuronal constituents could arise from transsynaptic neuronal degeneration. Anterograde transsynaptic damage from an optic nerve injury leading to neuronal loss in the LGN has been described in MS, [86], glaucoma [87], and after chemical injury to the optic nerve [88]. Recently retrograde transsynaptic degeneration has been interpreted from OCT manifestations of RNFL layer thinning in patients who suffered injury to the posterior visual pathways [89]. Further investigations are needed to validate the phenomenon of retrograde transsynaptic neuronal degeneration in the AVP, which could inform our understanding of mechanisms underpinning diffuse axonal loss in MS (distal from remote or active sites of inflammation) and add a further element of discussion to the “Inside-Out” versus “Outside-In” debate.

Interrogating the retina for evidence of MS-related pathology has prompted recent interest in the observation of microcystic macular edema (MMO) in the inner nuclear layer, which has been reported to occur in 5 to 6% of MS patients [84, 90]. First proposed by Gelfand and colleagues [90], MMO refers to retinal microcysts, which are thought to be a sign of inflammation in the retina [84, 90]. A follow-up study with OCT reported that increased inner nuclear layer thickness was associated with the development of MRI-measured contrast-enhancing lesions, new T2 lesions, and disability progression in MS patients [84]. Thus, inner nuclear layer thickness may be a structure marker of retinal inflammation that can be correlated with global metrics of disease activity in MS patients.

## 7. Future Directions

The AVP model offers an exciting opportunity to explore disease mechanisms that contribute to neurological disability in MS patients. Monitoring the acute and chronic consequences of clinically overt ON may shed light on factors that govern injury and repair after an inflammatory relapse in the CNS. Furthermore, longitudinal studies of MS patients unaffected by clinical ON events may help us determine whether axonal and neuronal damage occur independently of CNS inflammation in the AVP. Because the AVP model is amenable to multiple interrogation techniques, it may be possible to identify neuroprotective, remyelinating, and regenerative effects of emerging therapies being trialed in MS patients. Sensitive and standardized tests of vision can be compared with high resolution imaging measures of structural integrity in the AVP model to develop a structural-functional paradigm of CNS injury.
